# Dakin’s Solution and Dakin’s Oil in the Normal Peritoneal Cavity of the Dog

**Published:** 1919

**Authors:** 


***Dakin's Solution and Dakin's Oil in the Normal Peritoneal Cavity of the Dog.*** By Ernest G. GREY. *Johns Hopkins Hospital Bulletin*, October 1918.

The remarkable results obtained in resorting to normal solutions of chlorinated soda and dichloramine-T suggested the use of these agents in certain infections of the pleural and peritoneal cavities. Both solutions may be used with safety in the treatment of empyema, but should be employed with great caution in intra-abdominal infections.

Laboratory experiments were carried out to study the reaction of animals injected with an antiseptic under varying conditions. But the conclusions drawn from these experiments must be applied to clinical cases with a considerable amount of reservation, since it is most difficult to reproduce the same pathological process in animals as in patients.

In the first series a solution of chlorazene was injected into the peritoneal cavity of healthy dogs with a Record syringe. One animal received 20 cc. of a 1 per cent. solution, and died eleven days later; at necropsy the abdomen was found to be full of fluid, and fibrin was present in large amounts.

In another dog 90 cc. of a 1 per cent. solution were injected intraperitoneally. Six days later the abdomen was opened and 250 cc. of fluid removed. Thanks to this operation the animal made a rapid recovery, and 16 days later the viscera appeared quite normal.

In the second series Dakin's solution was experimented. Less than 30 cc. did not cause death, while larger amounts of fluid injected always proved fatal. An injection of a few cubic centimeters only, performed without anesthesia, caused severe abdominal pain and extreme restlessness.

In a third series, Dakin's solution was injected into the gall-bladder, either by means of a puncture or by transplanting a piece of jejunum in such a way that one end opened into the gall-bladder and the other upon the surface of the abdomen. No deleterious effects were ever noted.

A fourth series of experiments was carried out recently with Dakin's oil. One dog received 11 cc. intraperitoneally, and gave evidence of much abdominal discomfort with anorexia and some vomiting. It died 26 days after the injection, and postmortem examination disclosed an active former inflammatory process and an old inspissated abscess in the vicinity of the site of the injection.

Another dog received 20 cc. of oil into its peritoneal cavity and died on the following night. Besides an extensive peritonitis a perforation had occurred with ragged necrotic edges on the anterior surface of the stomach.

Following the injection of the oil, the gall-bladder became shrunken and contained some thick, yellowish mucus, though the remainder of the biliary tract showed no changes.

Finally Dakin's oil was injected into the normal pleural cavity, and led to a rapid death when no anesthesia was used, while no unusual symptoms marked convalescence when the animal was anesthetized.

*Conclusions.* These experiments suggest that the wall of an abscess cavity or sinus must play an important part in protecting the peritoneum from the chlorin when used successfully in certain abdominal infections. The maintenance of an adequate drainage tract seems also to be most important. Both chlorin antiseptics can only be resorted to in carefully selected cases.

